# Efficacy of adrenal venous sampling is increased by point of care cortisol analysis

**DOI:** 10.1530/EC-13-0063

**Published:** 2013-11-18

**Authors:** Kristin Viste, Marianne A Grytaas, Melissa D Jørstad, Dag E Jøssang, Eivind N Høyden, Solveig S Fotland, Dag K Jensen, Kristian Løvås, Hrafnkell Thordarson, Bjørg Almås, Gunnar Mellgren

**Affiliations:** 1 Hormone LaboratoryHaukeland University Hospital5021, BergenNorway; 2 Department of MedicineHaukeland University Hospital5021, BergenNorway; 3 Department of Clinical ScienceUniversity of Bergen5021, BergenNorway; 4 Department of RadiologyHaukeland University Hospital5021, BergenNorway

**Keywords:** adrenal, cardiovascular

## Abstract

Primary aldosteronism (PA) is a common cause of secondary hypertension and is caused by unilateral or bilateral adrenal disease. Treatment options depend on whether the disease is lateralized or not, which is preferably evaluated with selective adrenal venous sampling (AVS). This procedure is technically challenging, and obtaining representative samples from the adrenal veins can prove difficult. Unsuccessful AVS procedures often require reexamination. Analysis of cortisol during the procedure may enhance the success rate. We invited 21 consecutive patients to participate in a study with intra-procedural point of care cortisol analysis. When this assay showed nonrepresentative sampling, new samples were drawn after redirection of the catheter. The study patients were compared using the 21 previous procedures. The intra-procedural cortisol assay increased the success rate from 10/21 patients in the historical cohort to 17/21 patients in the study group. In four of the 17 successful procedures, repeated samples needed to be drawn. Successful sampling at first attempt improved from the first seven to the last seven study patients. Point of care cortisol analysis during AVS improves success rate and reduces the need for reexaminations, in accordance with previous studies. Successful AVS is crucial when deciding which patients with PA will benefit from surgical treatment.

## Introduction

Primary aldosteronism (PA) is a common cause of secondary hypertension; the prevalence is 2–15% in selected cohorts of hypertensive patients (1, 2, 3, 4, 5). Patients with PA have higher cardiovascular mortality and morbidity than controls with essential hypertension, possibly due to the presence of mineralocorticoid receptors in the heart and large vessels (2, 6). In ∼30–50% of the patients, the disease is unilateral, caused by for instance aldosterone-secreting adenomas, whereas the rest have bilateral disease (2, 6, 7, 8). The clinical management of PA depends on whether the disease is lateralized. Most patients with unilateral adenomas are either cured or have significant improvement of their hypertension after adrenalectomy (9). If the adrenal hypersecretion of aldosterone is bilateral, or a patient is unwilling to undergo surgery, medical treatment with a mineralocorticoid receptor antagonist is recommended (10).

Selective adrenal venous sampling (AVS) is the recommended method to determine whether aldosterone hyper production is lateralized (10). When recommended diagnostic cutoff values are used, the sensitivity and specificity for detecting unilateral disease are 95 and 100%, respectively. In comparison, adrenal computed tomography (CT) has sensitivity and specificity at 78% and 72–75% respectively (7, 11). During AVS, blood samples are drawn from the right and left adrenal veins as well as a peripheral vein. The ratio between cortisol measured in the samples taken from the putative adrenal veins and the peripheral blood sample is most commonly used to determine whether the sample is representative for adrenal vein blood (12). A recent study has suggested, however, that metanephrine might be a better marker of correct catheter placement (13). The procedure is technically challenging, and reported success rates range from 8 to 97% (11, 14, 15, 16). It is particularly demanding to obtain a representative sample from the right adrenal vein due to anatomic reasons (16). Although the left adrenal vein drains to the left renal vein, and hence is more easily identified, the right adrenal vein mostly drains directly into the vena cava and with a steep angle. The procedure might also be complicated by the collapse of the adrenal vein due to the gentle vacuum applied to obtain the sample.

In traditional AVS protocols, the cortisol levels in the blood samples are determined after the patient has left the examination room. Recently, four prospective studies have shown increased success rate when the sampling procedure was guided by intra-procedural rapid assay measurements of cortisol (17, 18, 19, 20). A retrospective study has showed that diagnostic centers that introduced such measurements when revising their AVS protocols had more improvement over time in success rates than diagnostic centers that did not (14). The published studies were all small, and used different study protocols. The cortisol gradient required to deem a sample representative of adrenal vein blood ranges from two- to fivefold. In the studies that used cosyntropin to stimulate cortisol production, the required cortisol gradient ranges from three- to fivefold. The number of procedures performed by each radiologist is variable, and different instruments have been used. Only one study analyzed cortisol using a point of care instrument (20). This study showed proof of concept of intra-procedural cortisol measurements, but included five patients and no control group. In the three studies including retrospective controls, the samples were analyzed in the main laboratory (17, 18, 19) or two patients were examined sequentially to minimize the total time used for the procedure (17, 18, 19).

We wanted to determine the success rate of AVS using intra-procedural, point of care cortisol analysis and compare with a historical AVS series, applying recommended criteria for sample selectivity (12).

## Subjects and methods

### Patients

Patients planned for AVS at the Department of Medicine, Haukeland University Hospital were sent invitation to participate in the study. The previous 21 AVS procedures were used as controls. All patients in the study period provided written consent. The study was approved by the Regional Ethics Committee for Medical Research (REK-Vest #2012-01856) and is registered at Clinicaltrials.gov with accession code NCT01761344. One patient experienced adverse effects after the procedure, and has provided written consent for this to be reported.

### Adrenal venous sampling

AVS was conducted sequentially under continuous cosyntropin infusion at a dose of 50 μg/h. The infusion was started 30 min before the procedure was initiated. For most procedures a Simmons 2/3 catheter was used to draw samples from the left adrenal vein whereas Hook, Simmons 2/3, or Shepherds hook catheters were used to draw samples from the right adrenal vein. A side hole was made in some catheters to decrease the vacuum at the tip of the catheter, and a gentle vacuum was applied to draw samples. For some procedures, a 0.014 inch floppy tip wire was inserted to prevent the adrenal vein from collapsing due to the applied vacuum. One to five blood samples were drawn from the external iliac vein and the putative left and right adrenal veins. The samples were transferred to Li/Heparin tubes for the point of care cortisol assay and serum tubes for routine assays. The sheath was not removed before the results of the point of care cortisol assay were available. If the assay revealed that the samples were not representative, repeated samples were drawn. The procedure was terminated after the third attempt to obtain representative samples or when the radiologist terminated due to increased risk of complications.

### Intraoperative cortisol analysis

Cortisol was analyzed at point of care using the AIA-360 Cort-pac system (TOSOH Bioscience, Tokyo, Japan) according to the protocol provided by the manufacturer. The lithium/heparin tubes were centrifuged immediately at 4440 ***g*** for 2 min. The samples were analyzed for cortisol undiluted and at 1/20 and 1/39 dilution (in cortisol-free serum provided by the assay supplier). We used a cutoff ratio of 5:1 between adrenal venous sample and peripheral sample to determine whether the sample was representative for adrenal blood as described in reference (12).

### Routine analysis

Serum cortisol was analyzed using Immulite 2000 (Siemens, Erlangen, Germany) according to the supplier's protocol and an inhouse multisteroid LC-MS/MS assay (21). The Siemens Immulite system automatically dilutes samples at 1/5 if the signal exceeds 1380 nmol/l, and samples were analyzed at 1/10, 1/20, and 1/40 sequentially. For LC-MS/MS analysis, all samples were diluted 1/50 in steroid-depleted serum (SunnyLab, Sittingbourne, UK) using an automated pipetting system (Hamilton Microlab Star, Bondzau, Switzerland). Aldosterone was detected using a RIA assay (Coat-a-count Aldosterone, Siemens). As criteria for lateralization, we used aldosterone:cortisol ratio in adrenal venous sample on one-side four times greater than that on the other side (12).

### Statistical analysis

*P* values were determined by applying two-tailed Fisher's exact test, Student's *t*-test, or Wilcoxon's signed-rank test. A *P* value of <0.05 was considered statistically significant. Trend lines for biochemical correlations were fitted using linear regression.

## Results

### Patients

The clinical characteristics of the patients in the study cohort and controls are shown in Table 1. Notably, most of the patients had been diagnosed with hypertension several years before AVS was carried out. The prevalence of hypokalemia, as defined by serum potassium values below the reference limits or use of oral potassium supplements, was higher than what has been reported from several other diagnostic centers (6, 22, 23). Some patients had developed hypokalemia several years before the diagnosis of PA was made (not shown). Among the study patients, seven had previously undergone an unsuccessful AVS, the corresponding number in the historical series was three. The two groups were not significantly different with regard to age, ratio between women and men, and number of patients who had undergone an unsuccessful AVS previously. The relative contribution from the two radiologists who conducted the procedures was similar in the two groups.

### Adrenal venous sampling

**Figure 1:**
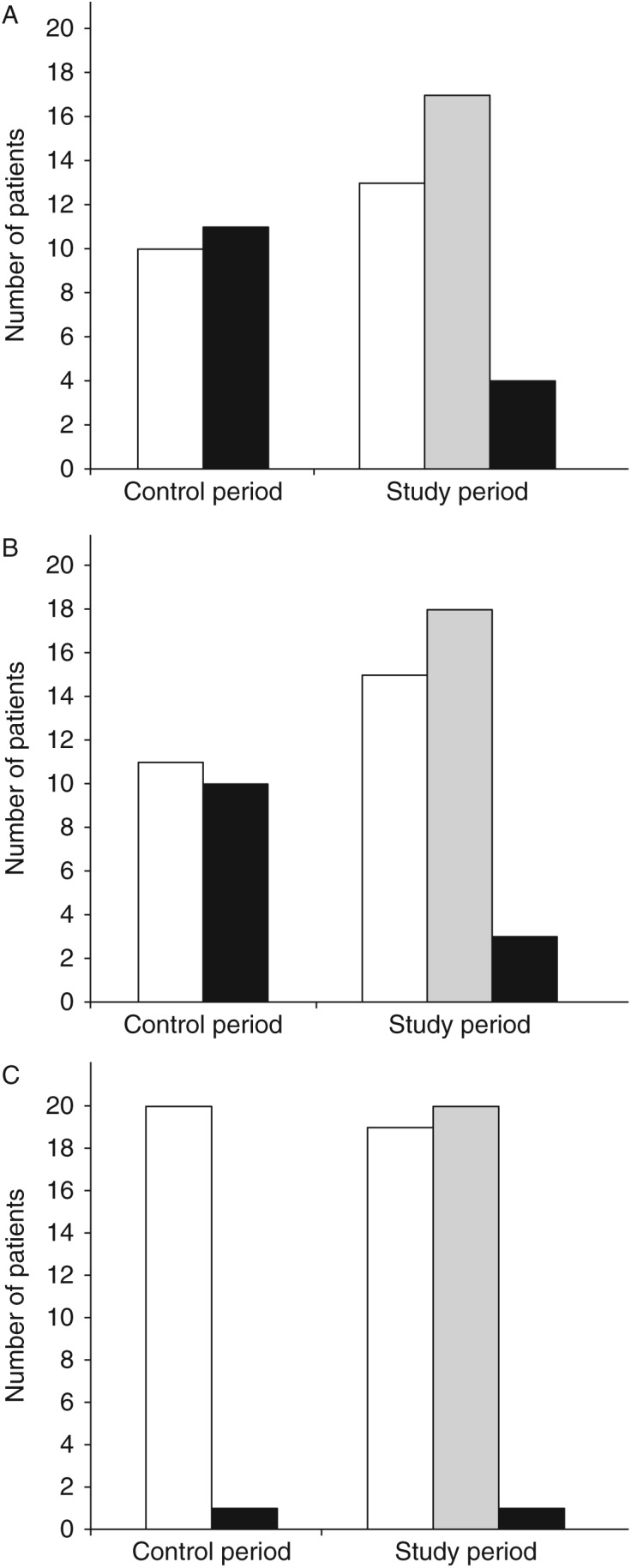
Number of patients whose first set of samples was representative for adrenal venous blood (white columns), representative samples were obtained before the procedure was terminated (gray columns) or representative samples were not obtained (black columns). (A) Data for both adrenal veins, (B and C) Data for right and left adrenal veins respectively.

Representative adrenal venous samples were obtained bilaterally in 17 of the 21 study patients (81%), whereas the procedure was successful in only ten of the 21 historical controls (48%) (Fig. 1A). The increased success rate was due to a significant increase in correct sampling from the right adrenal vein (Fig. 1B; *P*=0.0431). The success rate of left AVS was unchanged (Fig. 1C). The first set of samples was representative for 13 of the study patients, and the first resampling resulted in representative samples from four additional patients, for three of which resampling from the right adrenal vein was required. Of the four unsuccessful procedures, renewed sampling was done twice in two patients, whereas the procedure was terminated due to increased risk of complications after the second sampling in two of the patients. Seven of the study patients had previously undergone an unsuccessful procedure. The procedure was successful for six of these (not shown).

**Figure 2:**
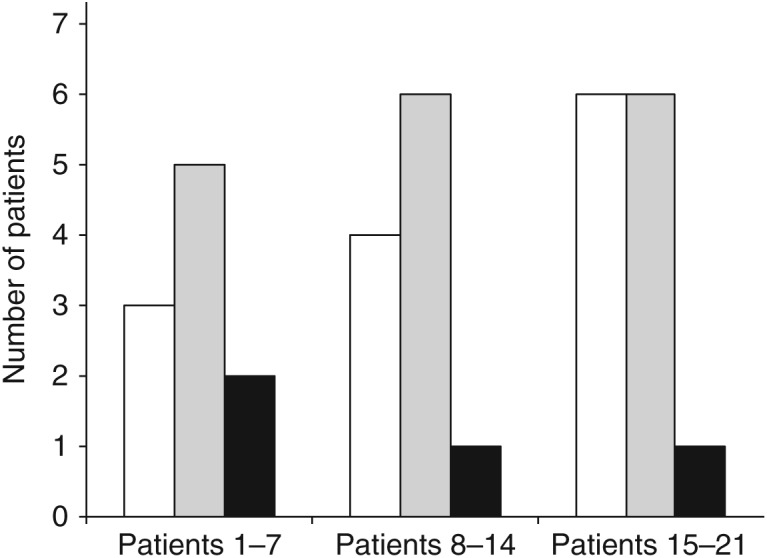
Number of patients whose first set of samples was representative for adrenal venous blood (white columns), representative samples were obtained before the procedure was terminated (gray columns), or where representative samples were not obtained (black columns) as a function of study number.

We also observed an increased success rate without repeated sampling throughout the study period. Whereas two of the seven first procedures were successful without renewed sampling, six of the final seven patients did not require repeated sampling (Fig. 2). The mean time from patient arrived the radiology suite until procedure was terminated was 138 min. The mean timespan before the first set of samples was drawn was 88 min (data not shown). In the procedures where the first set of samples was representative, the mean timespan from the acquisition of the last sample till the procedure was completed was 18 min. One of the patients in the study group was readmitted to hospital due to persistent pain. CT did not show ongoing bleeding, but a small amount of fluid was observed around a diffusely edematous right adrenal gland. The patient was treated conservatively, and was discharged without sequelae. No other adverse effects were noted in the study population or the historical series.

### Assay performance

**Figure 3:**
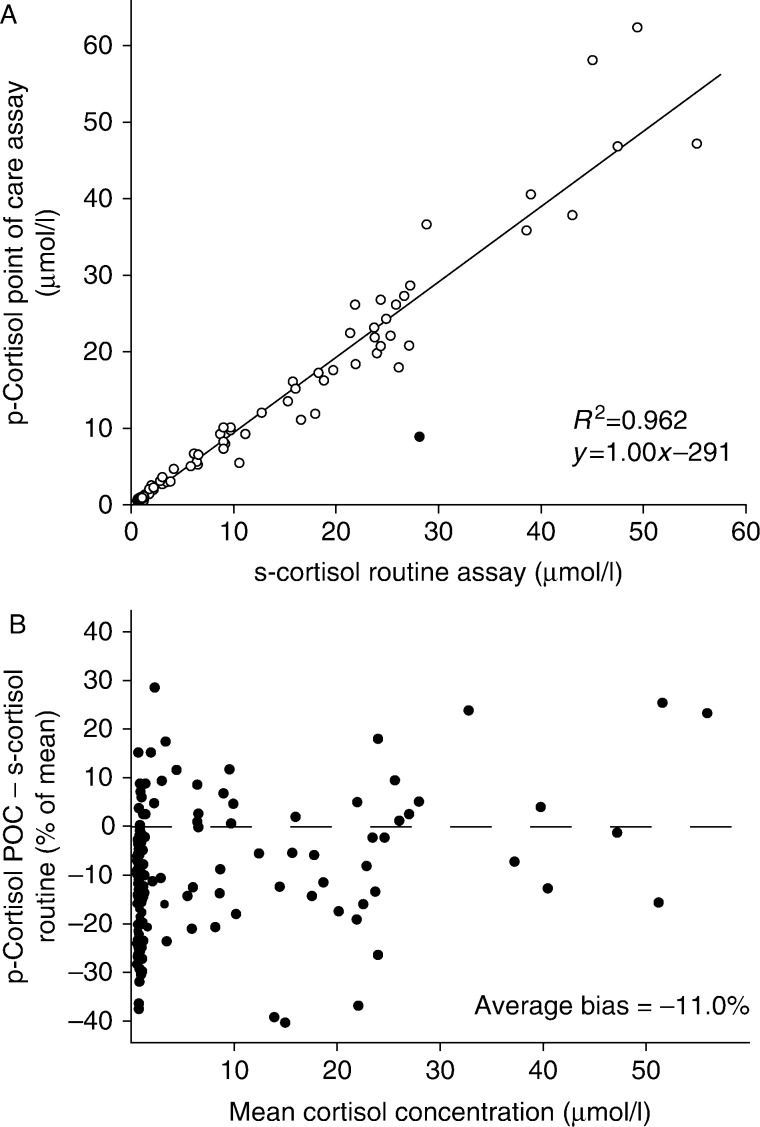
Comparison of results of point of care plasma cortisol and routine serum cortisol. (A) Correlation between p-cortisol in the point of care assay and s-cortisol in the routine assay. Open circles were included in the correlation analysis, closed circles were considered outliers. (B) Difference between the two assays as a percentage of the mean cortisol concentration.

The technical specifications of the point of care cortisol assay used during the AVS procedures are presented in the Supplementary Section, see section on supplementary data given at the end of this article. Briefly, the cortisol concentrations measured by the intraoperative cortisol assay correlated well with that obtained using a routine immunological cortisol assay (Fig. 3 and Supplementary Figure 1C–E, see section on supplementary data given at the end of this article) or an LC-MS/MS protocol (Supplementary Figure 1F). The average bias between the routine immunological assay and the rapid cortisol assay was −11%.

## Discussion

We found that after implementation of intra-procedural point of care cortisol analysis, the success rate of AVS procedures increased from 48% in the control period to 81% in the study period. This was due to an increase in the success rate of right AVS. There was a trend toward increased number of successful procedures without the need of resampling over the study period. The increased success rate can be attributed both to the opportunity to draw a new set of samples, as was done successfully in four patients, as well as a training effect for the radiologists. All previously published studies have shown significant increased success rate after repeated sampling (Table 2) (17, 18, 19). The trend toward increased success of initial sampling throughout the study period reproduces the results of Betz *et al*. (19). In our study, there was, however, a higher proportion of patients undergoing their second AVS among the first seven of the study patients. The uneven distribution of these patients might constitute a confounder to the observed training effect. However, there was no significant difference between the successful sampling of patients undergoing first and repeated procedures (not shown). We therefore find this to be an unlikely confounder. It is difficult to differentiate between the training effect due to shorter time interval between procedures in the study period and the effect of the point of care cortisol analysis. This also applies to the study by Betz *et al*. (19), where the intervention group was examined over a shorter time period than the controls. A shorter time span between procedures may thus represent a potential confounder in both studies.

Of the previously published studies on intra-procedural cortisol measurements during AVS, only the study reported by Rossi *et al*. (17) required a fivefold cortisol gradient for successful sampling. In that study, a single radiologist performed all 25 AVS. In the current study, all the 21 procedures were performed by two radiologists. We used an approach where the length of the procedure was kept at a minimum, and the patient was not moved to recovery before resampling, as described in two previous studies (17, 18). This approach was chosen to limit patient discomfort. Despite this, the radiology suite was only occupied for a mean 50 min after the first set of samples was drawn, 18 min if the first set of samples was representative. In comparison, Rossi *et al*. used 200 min to examine two patients if both procedures were successful.

Although there are no randomized trials showing effect of intra-procedural cortisol assay during AVS, four prospective studies, including this study, have shown increased success rate upon implementation of cortisol measurements (Table 2) (17, 18, 19). The increased success rate is independent of whether cosyntropin is used, the cortisol gradient required to deem the sample representative, the assay employed, and the number of procedures conducted by the individual radiologist in the study period. No published trials showing negative results have been identified. In total, including this study, two patients have had serious complications and required prolonged hospital stay or readmission to hospital. Neither patient has experienced further sequelae. Of the 123 patients subjected to AVS with intra-procedural cortisol analysis, this gives a complication rate of 1.6%, which is not significantly higher than what is reported for the procedure in general (7).

All published prospective studies are from centers with intermediate initial success rates (range 48–73%). The accumulated evidence for utilizing intra-procedural cortisol assays during AVS at centers with success rates <75% is compelling. Implementing the assay increases the quality of patient care by reducing the need for repeated procedures. The rate of complications is not significantly increased compared to protocols without point of care cortisol measurements.

## Supplementary data

This is linked to the online version of the paper at http://dx.doi.org/10.1530/EC-13-0063.

## Figures and Tables

**Table 1:** Clinical and procedure characteristics of patients examined with and without point of care cortisol analysis.

	**Historical controls**	**Study population**
Women/men	7/14	9/12
Radiologist 1/2	12/9	8/13
Patients with previous nonrepresentative AVS	3	7
Age (years) (median and range)	54 (39–77)	55 (30–69)
Days since the radiologist's previous procedure (median and range)	28 (2–147)	7 (1–110)
Samples taken at primary attempt (median and range)	4 (3–6)	5 (4–9)
Months of known hypertension (median and number of patients)	96 (*n*=15)	150 (*n*=19)
Patients with hypokalemia^a^ (%)	76	81
Blood pressure at admission (median)	164/102	162/101
Number of antihypertensive used^b^ (mean and range)	3 (2–5)	3 (1–6)
Lateralized/bilateral disease	6/4	13/4

a Patients were considered hypokalemic if they used potassium supplements and/or had a blood potassium level below the reference range upon admission to AVS.

b Number of antihypertensive used before drug adjustment for procedure.

**Table 2:** Prospective studies of intra-procedural cortisol analysis during AVS. The cortisol selectivity ratio is the ratio of cortisol between adrenal vein sample and peripheral sample required to deem the samples representative.

**First author/reference**	**Historical control** (success/total)	**First set of samples** (success/total)	**After repeated samples** (success/total)	**Use of ACTH**	**Cortisol selectivity ratio**	**Assay**	**Number of radiologists**	**Serious complications** (historical/intervention)^a^
Betz (19)	26/47	25/46	39/46	No	>2	LIAISON-Kit	3	0/1
Rossi (17)	16/25	19/25	23/25	Yes	>5	LIAISON-Kit	1	0/0
Auchus (18)	22/30	27/30	29/30	Yes	>3	Avida Centaur	5	0/0
Presented study	10/21	13/21	17/21	Yes	>5	Tosoh AIA 360	2	0/1
Total	74/123	84/122	108/122					0/2

a Complications are considered serious if they required re-admission to hospital or prolonged hospitalization.
